# Supplementary material for: Use of antimicrobials during the COVID-19 pandemic: A qualitative study among stakeholders in Nepal
Source: PLOS Glob Public Health. 2023 Nov 14;3(11):e0002166. doi: 10.1371/journal.pgph.0002166 (PMC10645294; doi:10.1371/journal.pgph.0002166)
Supplement: S2 File — (PDF) [file pgph.0002166.s002.pdf]

1. APPENDIX I: Interview guide for dispensers

SSI number:	Location:

- Use this interview guide for interviews with dispensers (drug store staff/pharmacists/dispensers)
- Take notes during or after the interview for each topic – just the main points (in English)
- Include description of general ambience, non-verbal reactions and the rough degree of their emotions on particular topics
- You can change the questions but ensure that each issue is covered.
- Avoid using closed questions and probe frequently.
- **Obtain demographic data in the sheet below.**

**SSI Note Takers Sheet**					
**Note Takers initials:**					
**Date: DD-MM-YYY**					
**Dispensary location (town/settlement/neighbourhood):**					
**Socio-demographics**					
**Interviewer ID**	**Age**	**Sex**	**Education (years)**	**Remarks**	**Respondent ID**

1) Explain the study and ask for consent (memorise the verbal consent script as best you can), then record consent when you turn on microphone

e.g "Thank you for seeing me today…are you happy to take part in this study?

Turn the recorder on

Ok, so I have turned the microphone on. I just wanted to ask again, are you happy to take part in this study by speaking with me today?

2) Explore the themes below:

**OTC: (Definition: Buying "over the counter medicine" means purchasing medicine from a local dispenser who is not the health worker/physician or buying medicine without a prescription)**	
**Topic**	**Suggested questions/probes**
**Tell me about this dispensary…**	• How many customers do you see in a day?<br>• Is the place yours or do you work for someone?<br>• How long have you been working here?<br>• How long has the shop been open?<br>• Have you seen any changes in this time? What are they?
**Main illnesses treated**	• What complaints do your customers suffer?<br>• Has this changed since the start of the pandemic?<br>• What type of medicines do your customers request?
**Demand for medicines**	• In general, what are the medication patient spontaneously demand?<br>• What is your general reaction to them?<br>• Do different types of people ask for different medicines?<br>• Do they directly ask for a medicine or is there a more subtle way of asking…e.g. telling a story about friend who was sick and received a certain medicine?<br>• Do you ever ask advice from anyone if you receive these requests?<br>• Has this changed since the start of the pandemic? In what ways?

**Treating respiratory infections**	• When a patient visits you with a cough, can you tell us what do you do?<br>• Do you do lab examination?<br>• Do you consult with someone else?<br>• Do you prescribe antibiotics?<br>• On what basis? when do you call for the follow up?) do patients with fever demand antibiotics?/if so, what do you do?<br>• What about common cold/flu, how do you treat them? Do these patients with flu demand for anitbiotics? If so, what do you do?
**Purchasing antibiotics**	• Do you dispense antibiotics?<br>• How often do you dispense antibiotics?<br>• What types of antibiotics?<br>• Do you sell them as individual tablets or as a whole course?<br>• How much do they cost?<br>• Which antibiotic do you sell the most?<br>• What complaints are they sold for?<br>• Do you ever refuse to sell antibiotics?<br>• If you don't have them in stock can you get them from somewhere else?<br>• Do you ever refer customers to other providers?
**Treating COVID-19**	• What do you do if some visits who you suspect might have COVID-19?<br>• Can you explain what kind of signs and symptoms these patients came with?<br>• Had any of these people have a positive test result with them? Did you refer them for a test?

	• Have you ever dispensed medicines to some who you thought might have COVID?<br>• How many times in the last month?<br>• What do you dispense?<br>• Did your treatment involve antibiotics? If yes, what antibiotics did you offer them? Why do you choose those?<br>• Can you tell whether these antibiotics work against COVID-19? If so, can you explain us how they work or how they do not work?<br>• Do you give advice?<br>• Do you think there was a rise in patients attending your clinic due to the pandemic?
**Potential consequences of OTC dispensing for COVID**	• Did these antibiotics work for or against the COVID-19 cases?<br>• Do you think COVID-19 pandemic may have increased the sale of antibiotics?<br>• Do you think COVID-19 may have increased the antibiotic pressure or antibiotic resistance?
**CLOSING**<br><br>Summarise the key points from the interview	Do you have any questions or concerns you'd like to raise? Thank you.

2. APPENDIX II: Interview guide for patients who were diagnosed with COVID-19

**SSI number:**	**Location:**

- Use this interview guide for interviews with patients or their peers, and relatives
- Take notes during or after the interview for each topic – just the main points (in English)
- Include description of general ambience, non-verbal reactions, and the rough degree of their emotions on particular topics
- You can change the questions but ensure that each issue is covered.
- Avoid using closed questions and probe frequently.
- **Obtain demographic data in the sheet below.**

**SSI Note Takers Sheet**					
**Note Takers initials:**					
**Date: DD-MM-YYY**					
**Address of the location (town/settlement/neighbourhood):**					
**Socio-demographics**					
**Interviewer ID**	**Age**	**Sex**	**Education (years)**	**Remarks**	**Respondent ID**

1) Explain the study and ask for consent (memorise the verbal consent script as best you can), then record consent when you turn on microphone

e.g "Thank you for seeing me today…are you happy to take part in this study?

Turn the recorder on

Ok, so I have turned the microphone on. I just wanted to ask again, are you happy to take part in this study by speaking with me today?

2) Explore the themes below:

**OTC: (Definition: Buying "over the counter medicine" means purchasing medicine from a local dispenser who is not the health worker/physician or buying medicine without a prescription)**	
**Topic**	**Suggested questions/probes**
**Tell me about your general condition**	• How are you today?<br>• How has been the last few weeks in terms of your health?
**Febrile illnesses OTC**	• Can you share me your experience if you have febrile illnesses recently?<br>• What did you do when you had fever?<br>• Did you seek medications from drug store?<br>• Do you know what medications you got for your fever?
**Demand for medicines**	• In general, what kind of medicine do you buy from the drug shop?<br>• How do you decide what medication to ask in the drug shop?<br>• Do you buy medicine for yourself or for your family members?<br>• Do you ever ask your relatives/neighbours for medicines?<br>• What about during the pandemic? Did you seek anyone's help to buy medicines?

**Treating respiratory infections**	• When you have a cough, can you tell us what do you do?<br>• Do you do lab test for your condition?<br>• Do you self-medicate with any medicines or antibiotics?<br>• What about common cold/flu, what do you do to cure it? Do you take medicines when you have flu? If so, what do you do?
**Purchasing antibiotics**	• Do you buy specific medicines such as antibiotics?<br>• How often do you buy antibiotics?<br>• What types of antibiotics do you buy?<br>• Do you generally buy medicine for full course or for only few days?<br>• How much do they cost?<br>• Which antibiotic do you buy the most?<br>• What conditions do you often buy the antibiotics for?<br>• Do you ever think not to buy antibiotics? Why?
**COVID-19 and OTC**<br>**(Perceived/projected attitude when a person gets COVID-19)**	• Have you seen/noticed any of your relatives/peers/friends who suffered from COVID-19?<br>• Based on your observation/experience, can you explain us how the symptoms and signs of COVID-19 begin and progress?<br>• Do you think all patients with COVID-19 seem to suspect or know their disease?<br>• Do these patients want to know their condition by lab confirmation? (if not why?)<br>• What do these patients with symptoms and signs of COVID-19 do to mitigate the severity?<br>• Do you think these patients buy medication over the counter?<br>• What kind of medications do they buy from the drug shops?

	• Do you think they receive antibiotics for their symptoms?
**COVID-19 and OTC (lived experience)**	• Before we talk about COVID-19 diagnosis, can you explain me what were the initial symptoms and signs and how it progressed?<br>• What did you do to mitigate the initial signs/symptoms?<br>• Before the test, did you suspect that it could be COVID-19?<br>• So, how did you decide that you would want to do the test?<br>• Initially and for the mild symptoms, what medications did you use? Where did you get them from? Can you explain, how did you get them?<br>• Do you know what medicine the drug shop provided to you? were they antibiotics?
**Potential consequences of OTC dispensing for COVID**	• Do you think receiving antibiotics from over the counter without diagnosis of COVID-19 helpful?<br>• What are the benefits and disadvantages of OTC medications for patients who are suspected/diagnosed with COVID-19?<br>• Do you think antibiotics work for or against the COVID-19 cases?<br>• Do you think COVID-19 pandemic may have increased the demands for antibiotics?<br>• Do you think COVID-19 may have increased the antibiotic pressure or antibiotic resistance?
**CLOSING**<br>Summarise the key points from the interview	Do you have any questions or concerns you'd like to raise? Thank you.

3. APPENDIX III: Interview guide for health workers

SSI number:	Location:

- Use this interview guide for interviews with health workers (clinicians who are responsible for the treatment of COVID-19)
- Take notes during or after the interview for each topic – just the main points (in English)
- Include description of general ambience, non-verbal reactions and the rough degree of their emotions on particular topics
- You can change the questions but ensure that each issue is covered.
- Avoid using closed questions and probe frequently.
- **Obtain demographic data in the sheet below.**

**SSI Note Takers Sheet**					
**Note Takers initials:**					
**Date: DD-MM-YYY**					
**Dispensary location (town/settlement/neighbourhood):**					
**Socio-demographics**					
**Interviewer ID**	**Age**	**Sex**	**Education (years)**	**Remarks**	**Respondent ID**

1) Explain the study and ask for consent (memorise the verbal consent script as best you can), then record consent when you turn on microphone

e.g "Thank you for seeing me today…are you happy to take part in this study?

Turn the recorder on

Ok, so I have turned the microphone on. I just wanted to ask again, are you happy to take part in this study by speaking with me today?

2) Explore the themes below:

**OTC: (Definition: Buying "over the counter medicine" means purchasing medicine from a local dispenser who is not the health worker/physician or buying medicine without a prescription)**	
**Topic**	**Suggested questions/probes**
**Tell me about your setting**	• How many patients do you see in a day?<br>• How long have you been working here?<br>• How long has the clinics/hospital been open?<br>• Have you seen any changes in this time? What are they?
**Main illnesses treated**	• What complaints do your patients suffer?<br>• Has this changed since the start of the pandemic?<br>• What type of medicines do your patients request?
**Demand for medicines**	• In general, what are the medication patient spontaneously demand?<br>• What is your general reaction to them?<br>• Do different types of patients ask for different medicines?<br>• Has this changed since the start of the pandemic? In what way?

**Treating respiratory infections**	• When a patient visits you with a cough, can you tell us what do you do?<br>• Do you do lab examination?<br>• Do you consult with someone else?<br>• Do you prescribe antibiotics?<br>• On what basis? when do you call for the follow up?) do patients with fever demand antibiotics?/if so, what do you do?<br>• What about common cold/flu, how do you treat them? Do these patients with flu demand for anitbiotics? If so, what do you do?
**Purchasing antibiotics**	• Do you think patients buy antibiotics over the counter?<br>• What types of antibiotics?<br>• Do dispensers sell them as individual tablets or as a whole course?<br>• How much do they cost?<br>• Which antibiotic do you think they sell the most?<br>• What complaints are they sold for?<br>• Do you think they ever refuse to sell antibiotics?
**Treating COVID-19**	• What do you do if someone who you suspect might have COVID-19?<br>• Can you explain what kind of signs and symptoms these patients came with?<br>• Had any of these people have a positive test result with them? Did you refer them for a test?<br>• How many times in the last month?<br>• Did your treatment involve antibiotics? If yes, what antibiotics did you offer them? Why do you choose those?

	• Can you tell whether these antibiotics work against COVID-19? If so, can you explain us how they work or how they do not work?<br>• Do you give advice?<br>• Do you think there was a rise in patients attending your clinic due to the pandemic?
**Potential consequences of OTC dispensing for COVID**	• Did these antibiotics work for or against the COVID-19 cases?<br>• Do you think COVID-19 pandemic may have increased the sale of antibiotics?<br>• Do you think COVID-19 may have increased the antibiotic pressure or antibiotic resistance?
**CLOSING**<br>Summarise the key points from the interview	Do you have any questions or concerns you'd like to raise? Thank you.
