# Supplementary material for: Use of antimicrobials during the COVID-19 pandemic: A qualitative study among stakeholders in Nepal
Source: PLOS Glob Public Health. 2023 Nov 14;3(11):e0002166. doi: 10.1371/journal.pgph.0002166 (PMC10645294; doi:10.1371/journal.pgph.0002166)
Supplement: Attachment — Submitted filename: Covid-19 Qual study comments.docx [file pgph.0002166.s004.docx]

**Manuscript PGPH-D-23-01183 Review**

**Introduction**

Lines 78-84 – The authors have briefly mentioned what other studies in this particular area have done. Perhaps this could be expanded a little more to explicitly identify the gaps in knowledge surrounding this topic and why this study has been conducted? How does this study help to fill that gap?

**Methodology**

Line 117-126 – Is there a specific reason why 30 participants were selected?

**Results**

Line 108 – was asked instead of were asked

Line 212 – recall their names instead of name

Line 168 – A lot of the patients did not have knowledge about what constituted antibiotics and AMR

& Line 220-221 – Most of them thought that AMR might have increased due to pandemic

The two statements seem contradictory. How can the patients not have knowledge of AMR but think that it might have increased due to the pandemic?

Line 287 – repetition of ‘they’

Line 302 – two-thirds instead of two-third of total sales of antibiotics volume

Line 325 – scrutinization is not a word, perhaps ‘scrutiny’ would be appropriate

Line 347-348 – Second and third waves were deemed to mount pressure among clinicians as well to prescribe antibiotics

This needs to be rephrased.

Line 348-350 – The scenario was intense in rural regions where clinicians were under pressure to offer antibiotics because the patients travelled from far to attend the health facilities.

This study was conducted in an urban area. Was the scenario in rural areas mentioned by a clinician? If not a citation is needed.

**Discussion**

Line 440 – OTC-based instead of OTC based

Line 469-484 – patients’ practices of using home remedies provide an interesting insight into their thought processes and health seeking behaviour during the pandemic. However, such a detailed account might not be needed under the sub-title “OTC use of antibiotics during Covid-19”. Mention couple of sentences but an entire paragraph detailing what constitute for home remedies is not needed.

Line 483 – unevidenced is not a word

The authors also need to explain the implications of patients’, dispensers’ and clinicians’ practices might have on Nepal’s AMR crisis.

Regarding patients experience, the discussion briefly mentions self-medications, pressuring dispensers etc. but mainly focuses on home remedies. But the study’s focus is on antibiotics and AMR, so the authors need to expand bit more on those topics. For example, what are the implications of over-usage/over-prescription of antibiotics mentioned by the dispensers and clinicians?

Do the authors have suggestions for future research directions?
