# Supplementary material for: Use of antimicrobials during the COVID-19 pandemic: A qualitative study among stakeholders in Nepal
Source: PLOS Glob Public Health. 2023 Nov 14;3(11):e0002166. doi: 10.1371/journal.pgph.0002166 (PMC10645294; doi:10.1371/journal.pgph.0002166)
Supplement: Attachment — Submitted filename: RESPONSE TO REVIEWERS COMMENTS.docx [file pgph.0002166.s006.docx]

23rd September 2023

Dear Dr. Dinesh and reviewers

Thank you very much for your comments/suggestions and opportunity to revise our work. We have utilized your suggestions to improve our manuscript. Please see below our responses to your specific suggestions with corresponding changes in the manuscript.

We look forward to your kind consideration.

On behalf of all the authors,

Bipin Adhikari

PGPH-D-23-01183
Use of antimicrobials during the COVID-19 pandemic: a qualitative study among stakeholders in Nepal
PLOS Global Public Health

Dear Dr Bipin Adhikari,

Thank you for submitting your manuscript to PLOS Global Public Health. After careful consideration, we feel that it has merit but does not fully meet PLOS Global Public Health’s publication criteria as it currently stands. Therefore, we invite you to submit a revised version of the manuscript that addresses the points raised during the review process.

**Introduction**

**Lines 78-84 – The authors have briefly mentioned what other studies in this particular area have done. Perhaps this could be expanded a little more to explicitly identify the gaps in knowledge surrounding this topic and why this study has been conducted? How does this study help to fill that gap?**

**AUTHORS:** Thank you for the question. We appreciate your suggestion. We have revised the last paragraph in Introduction section to address the research gap and revised paragraph reads as follows:

With efforts and resources diverted towards the control and management of COVID-19 infections, the pandemic’s potential impact on the use of antimicrobials and potential rates of AMR has often been overlooked [21]. Given its potentially critical impact, few studies have sought to understand how the COVID-19 pandemic affected the use of antimicrobials. Previous studies from Nepal, found that around 70-98% of COVID patients were treated with antibiotics in the hospital [22-24]. Another study found more than half of patients admitted in intensive care unit (ICU) in a tertiary care hospital were prescribed empirical antimicrobials [25]. The use of antibiotics was widespread even when only around half of the COVID infections had bacterial co-infections, and among severe and moderate COVID patients, around 20-25% had antimicrobial resistance [26]. Nonetheless, no studies have used qualitative research methods to examine the underlying social, cultural and health system contributors of antimicrobial use during this period. Drawing on SSIs with diverse stakeholders, including patients, clinicians and drug dispensers, this article examines the use of antimicrobials, during the pandemic in Kathmandu, Nepal.

**Methodology**

**Line 117-126 – Is there a specific reason why 30 participants were selected?**

**AUTHORS:** Thank you for the question. We have added a statement to clarify the rationale for selection of 30 participants and that reads as follows:

A total of 5-10 participants were targeted for each category to allow the flexibility to adjust the number for data saturation.

**Results**

**Line 108 – was asked instead of were asked**

**AUTHORS:** Thank you for the suggestion. Revised as suggested.

**Line 212 – recall their names instead of name**

**AUTHORS:** Thank you for the suggestion. Revised as suggested.

**Line 168 – A lot of the patients did not have knowledge about what constituted antibiotics and AMR**

**& Line 220-221 – Most of them thought that AMR might have increased due to pandemic**

**The two statements seem contradictory. How can the patients not have knowledge of AMR but think that it might have increased due to the pandemic?**

**AUTHORS:** Thank you for the suggestion. Aware of these seemingly contradicting statements, when patients were probed (after explaining what AMR means) on potential implications of over-use of antimicrobials, patients thought that AMR **may have been** increased by pandemic. The latter statement implies a weak perceived association. We have revised the statement to reflect this and reads as follows:

When probed on possible implications of over-use of antimicrobials, patients thought that AMR might have increased due to pandemic.

**Line 287 – repetition of ‘they’**

**AUTHORS:** Thank you for the suggestion. Revised as suggested.

**Line 302 – two-thirds instead of two-third of total sales of antibiotics volume**

**AUTHORS:** Thank you for the suggestion. Revised as suggested.

**Line 325 – scrutinization is not a word, perhaps ‘scrutiny’ would be appropriate**

**AUTHORS:** Thank you for the suggestion. Revised as suggested.

**Line 347-348 – Second and third waves were deemed to mount pressure among clinicians as well to prescribe antibiotics**

**This needs to be rephrased.**

**AUTHORS:** Thank you for the suggestion. Statement rephrased as

Second and third waves were deemed to mount pressure among clinicians to prescribe antibiotics.

**Line 348-350 – The scenario was intense in rural regions where clinicians were under pressure to offer antibiotics because the patients travelled from far to attend the health facilities.**

**This study was conducted in an urban area. Was the scenario in rural areas mentioned by a clinician? If not a citation is needed.**

**AUTHORS:** Thank you for the comment. This was a reflection by a clinician on his experience in rural regions how patients travelled from far to reach out a health worker and were unlikely to follow-up. In such circumstances, clinicians were in pressure to offer antibiotics so that patient can recover without requiring to follow-up.

**Discussion**

**Line 440 – OTC-based instead of OTC based**

**AUTHORS:** Thank you for the suggestion. Revised as suggested.

**Line 469-484 – patients’ practices of using home remedies provide an interesting insight into their thought processes and health seeking behaviour during the pandemic. However, such a detailed account might not be needed under the sub-title “OTC use of antibiotics during Covid-19”. Mention couple of sentences but an entire paragraph detailing what constitute for home remedies is not needed.**

**AUTHORS:** Thank you for the suggestion. While it’s true that the paragraph appears to divulge on health (treatment) seeking behavior, the impact of such behavior did have widespread impact on OTC use of antimicrobials. In fact, OTC use of antimicrobials are inherent in treatment seeking behavior, and why and how treatment seeking behavior was affected by COVID-19 is our central research question. We have shortened the paragraph as suggested and reads as follows:

Patients in this study followed home remedies and OTC medicines before they sought medical attention in hospitals. Our finding has been corroborated by several other studies from Asian and African countries [39-41]. Private pharmacies and dispensers comprised the larger chunk of informal prescription and sales of OTC medicines to these patients; this finding echoes with several previous reports [42-44]. Other reasons behind informal prescriptions were patients’ self-medications and induced pressure on dispensers, which were often guided by their close-contact and social media. Similar compelling factors were also outlined in the previous studies [15, 45]. Self-medications with home-based remedies were also widely practiced in Nepal that mostly comprised boiled water with basil leaves, ginger and turmeric powders and such practices are prevalent around the globe [39-41, 46]. Use of home-based medications also may have distorted the early treatment seeking behavior in formal services. Self-medication was prevalent among medical students too, more than half of medical students were found to self-medicate using OTC purchase [60]. Most of the patients perceived lowered self-esteem and psychological crises in the course of infection as seen in other studies [35] nonetheless, promoting confidence by recommending wide variety of unproven medications including herbs and unlicensed products can deteriorate the clinical course of the illnesses ultimately affecting treatment seeking behavior [47].

**Line 483 – unevidenced is not a word**

**AUTHORS:** Thank you for the suggestion. The word ‘unevidenced’ has been removed.

**The authors also need to explain the implications of patients’, dispensers’ and clinicians’ practices might have on Nepal’s AMR crisis.**

**Regarding patients experience, the discussion briefly mentions self-medications, pressuring dispensers etc. but mainly focuses on home remedies. But the study’s focus is on antibiotics and AMR, so the authors need to expand bit more on those topics. For example, what are the implications of over-usage/over-prescription of antibiotics mentioned by the dispensers and clinicians?**

**AUTHORS:** Thank you for the suggestion. As above, the paragraph has been reduced and the relevance of the retained topics have been explained.

We appreciate your suggestions on implications of over-sage/over-prescription of antimicrobials. We have added a dedicated section (Implications for redressing OTC use of antimicrobials during pandemic) in the revised version that reads as follows:

## Implications for redressing OTC use of antimicrobials during pandemic

COVID-19 was a special circumstance that exerted extraordinary pressure on the OTC use of antimicrobials, and future pandemics are likely to reproduce the phenomenon. The first step in mitigating the excess use of antimicrobials can begin from being cognizant of its demands, followed by launching multi-pronged approaches to reduce its demands such as through stakeholder and community engagement [73]. As AMR has been established to be exacerbated by pandemics, pandemic preparedness programs should include strategies to reduce the use of antimicrobials [74]. Revised policies to counteract excess and inappropriate use of antimicrobials during pandemic should be incorporated in contingency plans. Finally, a robust surveillance system should be employed to monitor the use of antimicrobials and antimicrobial resistance which should also include regular audits of both prescription-based sales and OTC-based sales of antimicrobials [75] without disruption during pandemic.

**Do the authors have suggestions for future research directions?**

**AUTHORS:** Thank you for the question. We have added our suggestions for the future research directions under the section ‘Strengths and limitations’ and reads as follows:

Future studies can explore the quantitative analysis on use of antimicrobials during pandemic with larger samples including the factors affecting its use and potential mitigation strategies.

Please submit your revised manuscript by Oct 22 2023 11:59PM. If you will need more time than this to complete your revisions, please reply to this message or contact the journal office at globalpubhealth@plos.org. When you're ready to submit your revision, log on to https://www.editorialmanager.com/pgph/ and select the 'Submissions Needing Revision' folder to locate your manuscript file.

Please include the following items when submitting your revised manuscript:

- A rebuttal letter that responds to each point raised by the editor and reviewer(s). You should upload this letter as a separate file labeled 'Response to Reviewers'.
- A marked-up copy of your manuscript that highlights changes made to the original version. You should upload this as a separate file labeled 'Revised Manuscript with Track Changes'.
- An unmarked version of your revised paper without tracked changes. You should upload this as a separate file labeled 'Manuscript'.

Guidelines for resubmitting your figure files are available below the reviewer comments at the end of this letter.

We look forward to receiving your revised manuscript.

Kind regards,

Dinesh Bhandari, Ph.D.
Academic Editor
PLOS Global Public Health

Journal Requirements:

**1. Please review your reference list to ensure that it is complete and correct. If you have cited papers that have been retracted, please include the rationale for doing so in the manuscript text, or remove these references and replace them with relevant current references. Any changes to the reference list should be mentioned in the rebuttal letter that accompanies your revised manuscript. If you need to cite a retracted article, indicate the article’s retracted status in the References list and also include a citation and full reference for the retraction notice.**

**AUTHORS:** Thank you for the suggestion.

**2. Please include a complete copy of PLOS’ questionnaire on inclusivity in global research in your revised manuscript. Our policy for research in this area aims to improve transparency in the reporting of research performed outside of researchers’ own country or community. The policy applies to researchers who have travelled to a different country to conduct research, research with Indigenous populations or their lands, and research on cultural artefacts. The questionnaire can also be requested at the journal’s discretion for any other submissions, even if these conditions are not met.  Please find more information on the policy and a link to download a blank copy of the questionnaire here: https://journals.plos.org/globalpublichealth/s/best-practices-in-research-reporting. Please upload a completed version of your questionnaire as Supporting Information when you resubmit your manuscript.**

**AUTHORS:** Thank you for the suggestion. We have added an inclusivity questionnaire form in revised submission.

**3. Please amend your detailed Financial Disclosure statement. This is published with the article. It must therefore be completed in full sentences and contain the exact wording you wish to be published.**

**a. State the initials, alongside each funding source, of each author to receive each grant.
b. If any authors received a salary from any of your funders, please state which authors and which funders.**

**If you did not receive any funding for this study, please simply state: “The authors received no specific funding for this work.”**

**AUTHORS:** Thank you for the suggestion.

**4. Please provide separate figure files in .tif or .eps format only and remove any figures embedded in your manuscript file. Please also ensure all files are under our size limit of 10MB.**

**For more information about figure files please see our guidelines:**

**https://journals.plos.org/globalpublichealth/s/figures 
https://journals.plos.org/globalpublichealth/s/figures#loc-file-requirement**

**AUTHORS:** Thank you for the suggestion. We have added a Figure file as .Tiff.

Additional Editor Comments (if provided):


[Note: HTML markup is below. Please do not edit.]

Reviewers' comments:

Reviewer's Responses to Questions

**Comments to the Author**

1. Does this manuscript meet PLOS Global Public Health’s publication criteria? Is the manuscript technically sound, and do the data support the conclusions? The manuscript must describe methodologically and ethically rigorous research with conclusions that are appropriately drawn based on the data presented.

Reviewer #1: Partly

Reviewer #2: Yes

2. Has the statistical analysis been performed appropriately and rigorously?

Reviewer #1: N/A

Reviewer #2: N/A

3. Have the authors made all data underlying the findings in their manuscript fully available (please refer to the Data Availability Statement at the start of the manuscript PDF file)?

The PLOS Data policy requires authors to make all data underlying the findings described in their manuscript fully available without restriction, with rare exception. The data should be provided as part of the manuscript or its supporting information, or deposited to a public repository. For example, in addition to summary statistics, the data points behind means, medians and variance measures should be available. If there are restrictions on publicly sharing data—e.g. participant privacy or use of data from a third party—those must be specified.

Reviewer #1: Yes

Reviewer #2: Yes

4. Is the manuscript presented in an intelligible fashion and written in standard English?

PLOS Global Public Health does not copyedit accepted manuscripts, so the language in submitted articles must be clear, correct, and unambiguous. Any typographical or grammatical errors should be corrected at revision, so please note any specific errors here.

Reviewer #1: Yes

Reviewer #2: Yes

5. Review Comments to the Author

Please use the space provided to explain your answers to the questions above. You may also include additional comments for the author, including concerns about dual publication, research ethics, or publication ethics. (Please upload your review as an attachment if it exceeds 20,000 characters)

Reviewer #1: The authors did a good job explaining the factors that affected usage and prescription of antibiotics among patients, pharmacies, and clinicians in Nepal. In general, the study is important as it highlights some of the issues of global health importance like antimicrobial resistance and the need to understand the underlying drivers among people that exacerbates the current situation. I have included here comments that the authors can use to improve their manuscript.

**Reviewer #2: Discussion is not sufficient so the authors are requested to add more literature on discussion.**

**AUTHORS:** Thank you for the suggestion. We have extensively updated and revised the literature in both introduction and discussion sections.

**Authors are requested to explain the member checking process if they have done during this study and their reflexivity as well.**

**AUTHORS:** Thank you for the suggestion. We have reported on ‘member checking process’ following a CORE Q guideline and are aware of the pros and cons of its relevance (Birt 2016). We have reported on it under the section ‘data analysis’ that reads as follows:

The transcripts and the findings were not shared among the respondents for their opinion, although some of the preliminary findings were re-discussed among the investigators and were prioritized in the subsequent interviews.

In terms of reflexivity, we have added the details of the interviewer and data analyzer including the reflection sessions we had regularly for thematic synthesis and reads as follows:

Thematic analysis of the transcripts was conducted at various points of the study that entailed discussions of the findings and reflection among the interviewer (BD) and researchers (BA, KRR and SA). These core investigators are from Nepal and have living experience on OTC use of antimicrobials and have bearing on the interpretation of the data.

6. PLOS authors have the option to publish the peer review history of their article (what does this mean?). If published, this will include your full peer review and any attached files.


**Do you want your identity to be public for this peer review?** If you choose “no”, your identity will remain anonymous but your review may still be made public.


For information about this choice, including consent withdrawal, please see our Privacy Policy.

Reviewer #1: No

Reviewer #2: No
